# Rare Breast Cancer Histotypes—A Retrospective Study and Literature Review

**DOI:** 10.3390/jcm13030643

**Published:** 2024-01-23

**Authors:** Allan Hoi Kin Lam, Michael Tiong Hong Co, Ava Kwong

**Affiliations:** Division of Breast Surgery, Department of Surgery, The University of Hong Kong, Hong Kong, China; lhk886@ha.org.hk (A.H.K.L.); mcth@hku.hk (M.T.H.C.)

**Keywords:** breast cancer, rare breast cancer, oncology

## Abstract

Breast cancer is the most common cancer among women globally and can be classified according to various histological subtypes. Current treatment strategies are typically based on the cancer stage and molecular subtypes. This article aims to address the knowledge gap in the understanding of rare breast cancer. A retrospective study was conducted on 4393 breast cancer patients diagnosed from 1992 to 2012, focusing on five rare subtypes: mucinous, invasive lobular, papillary, mixed invasive and lobular, and pure tubular/cribriform carcinomas. Our analysis, supplemented by a literature review, compared patient characteristics, disease characteristics, and survival outcomes of rare breast cancer patients with invasive carcinoma (not otherwise specified (NOS)). Comparative analysis revealed no significant difference in overall survival rates between these rare cancers and the more common invasive carcinoma (NOS). However, mucinous, papillary, and tubular/cribriform carcinomas demonstrated better disease-specific survival. These subtypes presented with similar characteristics such as early detection, less nodal involvement, more hormonal receptor positivity, and less human epidermal growth factor receptor 2 (HER2) positivity. To conclude, our study demonstrated the diversity in the characteristics and prognosis of rare breast cancer histotypes. Future research should be carried out to investigate histotype-specific management and targeted therapies, given their distinct behavior.

## 1. Introduction

Breast cancer is the most common cancer in the female population worldwide [1]. It accounted for 28.5% of new cancers in females diagnosed in Hong Kong in 2021. The median age of diagnosis for breast cancer in our locality is 58 years old [2].

The World Health Organization’s fifth edition of the *Classification of Tumours of the Breast*, published in 2019, offers a comprehensive classification of breast cancers based on histology and genetic profiling [3]. The predominant subtype of breast cancer is the invasive type, often classified as ‘not otherwise specified (NOS)’ or ‘no special type (NST)’ as it does not exhibit the defining features of other more distinctive subtypes. In general, breast cancer management strategies are tailored according to cancer stage and molecular subtypes, including triple-negative, HER2-positive, and estrogen receptor (ER)-positive variations. Currently, there are limited guidelines for managing rarer tumor types.

This study aims to review the management and outcomes of rare breast tumor subtypes at a tertiary academic breast surgery unit in Hong Kong. It also includes a comprehensive literature review, covering current knowledge on the disease characteristics of these more commonly encountered rare breast tumors.

## 2. Materials and Methods

This study was approved by the ethical committee of the study center. Patient consent was obtained for data collection and analysis. The study was performed in line with the Declaration of Helsinki.

Patients diagnosed with invasive breast cancer between 1992 and 2012 were identified from a prospectively maintained database and selected for detailed analysis. The analysis was focused on the five most prevalent rare breast cancers—mucinous carcinoma, invasive lobular carcinoma, papillary carcinoma, mixed invasive (NOS) and lobular carcinoma, and pure tubular/cribriform carcinoma. A thorough literature search was conducted using an online literature database (PubMed^®^). The individual epidemiology, molecular biology, tumor behavior, and clinical outcome were summarized. Complementing this retrospective approach, the dataset supporting this study’s findings has been meticulously maintained to ensure accuracy and reliability. The dataset is available for independent verification and research purposes upon request from the corresponding author.

Invasive carcinoma (NOS) was identified as the most common invasive breast cancer (85.5%) amongst all the cancer types and was selected as the control group for further comparison with other rare breast cancer groups. Patient characteristics, tumor characteristics, overall survival, disease-free survival as well as breast cancer-related survival were analyzed.

Differences in the clinical data were analyzed using an independent-sample *t*-test and chi-squared test for *p*-values. Disease-free survival was defined from the date of surgery to the time of documented recurrence, including locoregional recurrence and/or distant metastasis. Survival curves were constructed using the Kaplan–Meier method. Statistical significance was defined as *p* < 0.05. All statistical analyses were performed using IBM SPSS Statistics version 24.0 (IBM Corp, Armonk, New York, NY, USA).

## 3. Results

In total, 4393 patients with invasive breast tumors were diagnosed in our center from 1992 to 2012 (Table 1). Of those, 4202 had undergone surgical resection, while 191 of them did not receive resection either due to advanced-stage disease or patient refusal. This group was excluded, and the surgical resection group was selected for further analysis.

The mean follow-up time for our patients was 112 months, with excellent overall compliance to follow-up (92.9%).

According to the results of our study, invasive carcinoma (NOS) was the most common breast cancer (pure variant) type (*n* = 3594, 85.5%). We further identified the top five rare breast cancers in Hong Kong, namely mucinous carcinoma (*n* = 141, 3.36%), invasive lobular carcinoma (*n* = 139, 3.31%), invasive papillary carcinoma (*n* = 82, 1.95%), mixed invasive (NOS) and lobular carcinoma (*n* = 67, 1.59%), and pure tubular/cribriform carcinoma (*n* = 45, 1.07%). Notably, 23% of the invasive lobular carcinomas were bilateral.

### 3.1. Patient Characteristics (Table 2)

The mean age of onset in invasive ductal carcinoma was 51.1 ± 12.7 years old. We observed an older age of onset in mucinous carcinoma (54.6 ± 16.3 years old, *p* = 0.013) and papillary carcinoma (58.2 ± 15.2 years old, *p* < 0.001). The mean follow-up time for all groups of patients was comparable, except in the mixed invasive carcinoma (NOS) and lobular carcinoma group, which was higher (*p* = 0.003). The overall compliance to follow-up was 92.8%, with the default rate ranging from 3.6% to 10.4%.

**Table 2:** Patient characteristics.

Characteristic	Invasive Carcinoma (NOS)	Mucinous	Invasive Lobular	Papillary	Mixed Invasive (NOS) and Lobular	Tubular/Cribriform
Number of Patients	3594	141	139	82	67	45
Age (years)	51.1 ± 12.7	54.6 ± 16.3 * (*p* = 0.013)	52.6 ± 11.6	58.2 ± 15.2 * (*p* < 0.001)	52.7 ± 12.7	54.5 ± 13.3
Follow-Up Time (months)	111.2 ± 65.4	115.7 ± 61.6	101.4 ± 58.7	115.2 ± 57.8	139.0 ± 74.2 * (*p* = 0.003)	137.5 ± 58.1
Default Rate	7.3%	3.6%	7.2%	7.3%	10.4%	8.9%

Note: Values are expressed in mean ± standard deviation. * *p* < 0.05, *p*-values obtained using independent-sample *t*-test with comparison to invasive carcinoma (NOS) group.

### 3.2. Disease Characteristics (Table 3)

The majority of the invasive carcinoma (NOS) and the five most common rare breast cancers presented with a palpable breast mass, ranging from 74.9% to 87.8%. The second most common mode of detection was screen-detected lesion by mammography and/or ultrasonography (2.4–13.3%), followed by incidental findings on either computed tomography scan or positron emission tomography (0–1.2%).

**Table 3:** Disease characteristics and treatment choice by histological type.

Characteristics	Invasive Carcinoma (NOS)	Mucinous	Invasive Lobular	Papillary	Mixed Invasive (NOS) and Lobular	Tubular/Cribriform
Mode of Detection						
Palpable breast mass	82.6%	84.4%	74.9%	87.8%	86.6%	77.8%
Screen-detected	6.0%	5.7%	12.2%	2.4%	4.5%	13.3%
Incidental (CT/PET-CT)	0.4%	0%	0.7%	1.2%	0%	0%
Staging						
Stage I	35.8%	46.1% * (*p* = 0.0125)	25.9% * (*p* = 0.0167)	37.8%	22.4% * (*p* = 0.0232)	68.9% * (*p* < 0.0001)
Stage II	39.7%	41.1%	38.1%	11.0% * (*p* < 0.001)	43.3%	24.4% * (*p* = 0.037)
Stage III	17.6%	5.7% * (*p* = 0.002)	30.9% * (*p* = 0.0001)	1.2% * (*p* = 0.0001)	25.4%	2.2% * (*p* = 0.0068)
Stage IV	3.1%	1.4%	2.2%	1.2%	4.5%	0%
Lymph node-positive	43.7%	12.8% * (*p* < 0.0001)	49.6%	3.7% * (*p* < 0.0001)	59.7% * (*p* = 0.009)	17.8% * (*p* = 0.005)
Mode of Surgery						
Breast conservative Therapy	25.2%	36.1%	16.6%	41.4%	20.9%	35.6%
Median tumor Size (cm)	2	2	2.5	1.3	2.5	1
Axillary Dissection						
Overall	81.7%	74.5%	79.1%	56.1%	92.5%	82.2%
2004 Onward	73.9%	60%	70.1%	50%	80%	66.7%
Recurrence						
Overall	9.5%	6.4%	9.4%	9.8%	4.5%	2.2%
Chest wall/breast	6.5%	5.7%	6.5%	8.5%	4.5%	2.2%
Axilla	3.0%	0.7%	1.4%	2.4%	0%	0%
Supraclavicular Fossa	2.1%	0%	3.6%	0%	0%	0%
Adjuvant Therapy						
Radiotherapy	56.1%	45.4% * (*p* = 0.0121)	59.0%	35.4% * (*p* = 0.0002)	58.2%	40.0% * (*p* = 0.0307)
Chemotherapy	55.8%	24.8% * (*p* < 0.0001)	54.0%	12.2% * (*p* = 0.0001)	53.7%	13.3% * (*p* < 0.0001)
Hormonal therapy	62.1%	78.0% * (*p* = 0.0001)	66.9%	43.9% * (*p* = 0.0001)	67.2%	66.7%
Targeted therapy	8.5%	1.4% * (*p* = 0.0026)	5.8%	0%	4.5%	0%

Notes: * *p* < 0.05, obtained using Pearson’s chi-squared test compared to the invasive carcinoma (NOS) group. Percentages are based on the total number of patients within each histological type.

Breast conservative therapy (BCT) was least commonly performed in invasive lobular carcinoma, (16.6% compared to 25.2% in invasive carcinoma (NOS)). This can be partly explained by the large median tumor size of invasive lobular carcinoma, which was the highest among the analyzed groups (2.5 cm).

Sentinel lymph node biopsy was first performed in our unit in 1996 on one patient, and it was followed by the formal launching of the procedure in 1999. In the current cohort, 1137 (25.9%) patients received sentinel lymph node biopsy, 633 (55.7%) of whom were positive for malignancy requiring subsequent axillary dissection.

The overall axillary dissection rate was 81.7% for invasive carcinoma (NOS). This rate was 74.5% for mucinous carcinoma, 79.1% for invasive lobular carcinoma, 56.1% for invasive papillary carcinoma, 92.5% for mixed invasive carcinoma (NOS) and lobular carcinoma, and 82.2% for tubular/cribriform carcinoma.

According to the results, three of the five most common rare breast cancers had significantly earlier-stage disease when compared with invasive carcinoma (NOS). Mucinous carcinoma mostly presented as Stage I disease (46.1% vs. 35.8%, *p* = 0.0125) and was less commonly seen as Stage III disease (5.7% vs. 17.6%, *p* = 0.0002). Papillary carcinoma mostly presented as Stage I disease (37.8%) and was less commonly seen as Stage II (11.0% vs. 39.7%, *p* < 0.0001) and Stage III disease (1.2% vs. 17.6%, *p* = 0.0001). Tubular/cribriform carcinoma was more commonly observed as Stage I disease (68.9% vs. 35.8%, *p* < 0.0001) and less commonly presented as Stage II (24.4% vs. 39.7%, *p* = 0.037) or Stage III disease (2.2% vs. 17.6%, *p* = 0.0068). They were also less likely to have nodal involvement when compared with invasive carcinoma (NOS) (43.7%), mucinous carcinoma (12.8%, *p* < 0.0001), papillary carcinoma (3.7%, *p* < 0.0001), and tubular/cribriform carcinoma (17.8%, *p* = 0.0005).

Mucinous carcinoma (ER: 69.4%, *p* < 0.0001; PR: 60.9%, *p* < 0.0001); mixed invasive carcinoma (NOS) and lobular carcinoma (ER: 73.1%, *p* = 0.0002; PR: 70.2%, *p* < 0.0001); and tubular/cribriform carcinoma (ER: 71.2%, *p* = 0.0049; PR: 66.8%, *p* = 0.0005) had higher estrogen receptor (ER) positivity and progesterone receptor (PR) positivity when compared with invasive carcinoma (NOS) (ER: 50.1%, PR: 40.9%).

Invasive lobular carcinoma only had higher estrogen receptor positivity (ER: 59.1%, *p* = 0.0373) compared with invasive carcinoma (NOS). In comparison, HER2 status was less likely to be positive in mucinous carcinoma (60.9%, *p* < 0.0001), invasive papillary carcinoma (4.9%, *p* = 0.0008), and mixed invasive carcinoma (NOS) and lobular carcinoma (3.0%, *p* = 0.0006), when compared with invasive carcinoma (NOS) (19.7%). Notably, 982 (22.4%) patients did not have ER/PR receptor status tested in the histopathology examination. Similarly, 1320 (30.0%) patients did not have HER2 status tested.

The overall or site-specific recurrence rate did not reach any statistical significance; however, it was more common to have locoregional (chest wall or breast) rather than axillary or supraclavicular recurrence.

### 3.3. Adjuvant Therapies (Table 3)

As for adjuvant therapy, radiotherapy, chemotherapy, hormonal therapy, and targeted therapy were common adjuncts after initial surgical resection. The rate of radiotherapy was significantly lower in mucinous (45.4%, *p* = 0.0121), papillary carcinoma (35.4%, *p* = 0.0002), and tubular/cribriform carcinoma (40.0%, *p* = 0.0307) compared with the control group (56.1%). These three groups were also reported to have a lower chemotherapy rate, with mucinous at 24.8% (*p* < 0.0001), papillary at 12.2% (*p* < 0.0001), and tubular/cribriform at 13.3% (*p* < 0.0001). Hormonal therapy was more commonly used in the mucinous group (78.0% vs. 62.1%, *p* = 0.0001) and less commonly used in the papillary group (43.9% vs. 62.1%, *p* = 0.0008), while targeted therapy was less commonly used in the mucinous group (1.4% vs. 8.5%, *p* = 0.0026).

### 3.4. Survival Analysis

**Figure 1:**
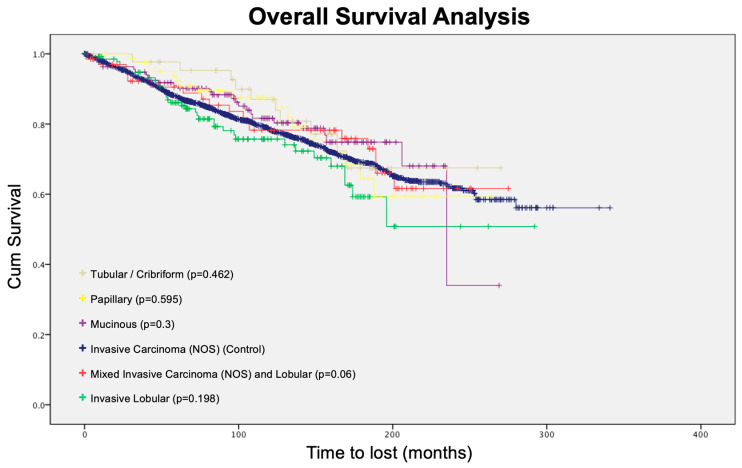
Overall survival analysis.

We conducted an overall survival analysis using invasive ductal carcinoma as a control group (Figure 1). However, none of the groups had a statistically significant difference in overall survival compared with the control group.

On further analysis, invasive carcinoma (NOS) and the five most common rare breast cancers demonstrated very favorable survival outcomes. The 5-year disease-free survival was 93.3%, and the 10-year disease-free survival was 91.5%. The 5-year overall survival was 89.6%, and the 10-year overall survival was 83.2%. Hence, the cause of death in the vast majority of patients who had surgical resection was very likely due to other diseases rather than breast cancer (Table 4).

**Figure 2:**
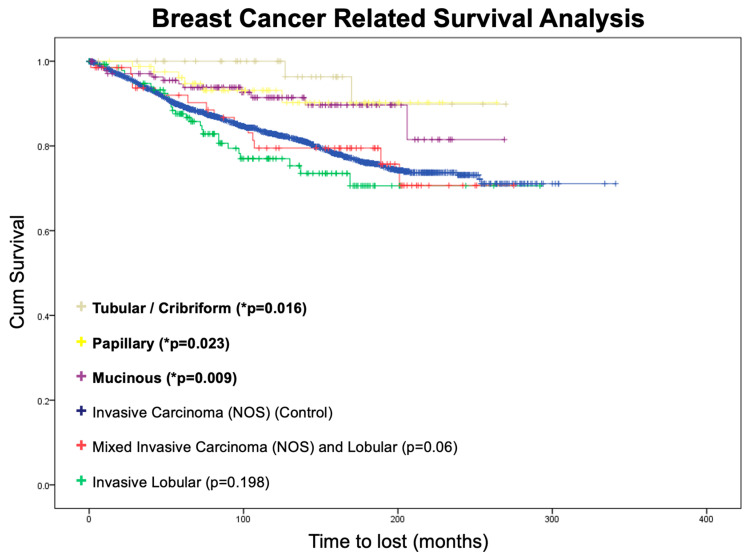
Breast cancer-related survival analysis. Note: * *p* < 0.05, obtained using the log-rank test, comparing individual Kaplan-Meier survival curves to the invasive carcinoma (NOS) group.

Breast cancer-related survival analysis was performed using the Kaplan–Meier method (Figure 2). Three groups of patients showed significantly better breast cancer-related survival when compared with invasive carcinoma (NOS) (i.e., the control group), namely mucinous carcinoma (*p* = 0.009), papillary carcinoma (*p* = 0.023), and pure tubular/cribriform carcinoma (*p* = 0.016).

## 4. Discussion

### 4.1. Literature Review

The literature search and review were performed using an online literature database (PubMed^®^) using the following keywords: breast carcinoma subtypes, disease characteristics, and rare breast cancers. Individual epidemiology, molecular biology, tumor behavior, and clinical outcomes are summarized below.

#### 4.1.1. Mucinous Carcinoma

Mucinous carcinoma is characterized by the production of abundant extracellular and/or intracellular mucin [4]. The median age at diagnosis is reported to be 71 years, with a range of 25 to 85 years [5]. This carcinoma is classified into two subtypes: the pure type, which is defined by mucin purity of 90% or more, and the mixed type, which has 50–90% mucin purity and includes an infiltrating ductal epithelial component [6].

The immunohistochemical profile of mucinous carcinoma typically shows positive hormonal receptor status, with estrogen receptor (ER) positivity at 94% and progesterone receptor (PR) positivity at 80%, while being HER2-negative [7,8,9]. The majority, 63.8%, are diagnosed with Stage T2. Mucinous carcinoma generally presents with a smaller tumor size and infrequent nodal involvement, ranging between 3% and 15% [6,10]. The disease-specific 5-year survival rate is reported to be 94%, and the 10-year survival rate is approximately 89% [11].

#### 4.1.2. Invasive Lobular Carcinoma

Invasive lobular carcinoma is characterized by the growth of small, round tumor cells in a distinctive single file or loose clusters pattern known as ‘Indian file’ [12]. The mean age at diagnosis is 63.4 ± 12.7 years [13]. The immunohistochemical profile typically shows positive hormone receptor status, with 92.7% estrogen receptor (ER) positivity, 67.4% progesterone receptor (PR) positivity, and a HER2 negativity rate of 89.3–93% [12,14,15].

This carcinoma subtype is frequently multicentric, with bilateral disease observed in 20–29% of cases [5]. It tends to metastasize in diverse patterns, often with less nodal involvement but a greater likelihood of spreading to the peritoneum, retroperitoneum, leptomeninges, gastrointestinal tract, uterus, and ovaries, as opposed to other histological types [5,12,16]. Due to its multicentric nature, breast conservative therapy (BCT) is performed less often, namely in 9.5% of cases compared to 12.7% in invasive ductal carcinoma (IDC) [11]. Disease-free survival rates for invasive lobular carcinoma are similar to IDC, with a 5-year DFS of 85.7% versus 83.5% for IDC (*p* = 0.13) [14].

#### 4.1.3. Papillary Carcinoma

Papillary carcinoma is characterized by cell proliferation around fibrovascular cores, typically forming a well-defined mass [17]. This type of cancer is most commonly diagnosed in postmenopausal women, with an average age at diagnosis of 65.7 ± 13.2 years [13]. It is categorized into various subtypes, including encapsulated (intracystic papillary carcinoma), solid papillary carcinoma, and invasive micropapillary carcinoma [17]. Hormonal receptor positivity is high in this carcinoma (ER + 100%, PR + 80%) [18], and it is predominantly HER2-negative [19].

The majority are identified at an early stage, with 45% at Stage I and 44% at Stage II. Only 3% present with distant metastasis, as found in a comprehensive review from the Netherlands that included 1073 papillary carcinoma cases [20]. The overall survival rate for this carcinoma is reported to be excellent, with 5-year survival up to 95% [17,20].

#### 4.1.4. Tubular/Cribriform Carcinoma

Tubular carcinoma is characterized by regular cells arranged in well-defined tubules, which are surrounded by an abundant fibro-hyaline stroma. It is categorized into two subtypes [21]: the pure type, where the tubular composition is 90% or more, and the mixed type, with tubular composition of at least 75%. The mean age at diagnosis is 57.0 ± 13.8 years [13]. The majority of cases express hormonal receptors, with 92.9% exhibiting estrogen receptor (ER) positivity and 87% showing progesterone receptor (PR) positivity, while 87.1% of them are HER2-negative [22]. This carcinoma is associated with a low incidence of lymph node metastasis (2–11%) and a low rate of local recurrence (1.43%) [22]. The overall prognosis is favorable, with a 5-year survival rate of 94%, even among patients with node-positive disease [23].

Cribriform carcinoma predominantly presents with a cribriform pattern in its invasive components. The median age at onset is 53 years old, with a range from 38 to 75 years old [24]. Subtypes include the classical type, which contains less than 50% tubular carcinoma component, and the mixed type, which includes 10% to 49% of other invasive carcinoma components, excluding tubular carcinoma [24]. The majority of cases express hormonal receptors, with 100% exhibiting estrogen receptor (ER) positivity and 69% showing progesterone receptor (PR) positivity [25]. HER2 negativity is observed in 98% of cases [26]. Nodal involvement is uncommon, at 14.3%, and distant metastasis is rare [27], contributing to an excellent 5-year overall survival rate of 90–100% [28].

#### 4.1.5. Mixed Ductal/Lobular Carcinoma

Multifocal disease occurs more frequently in patients with mixed ductal/lobular carcinoma, a condition characterized by the coexistence of ductal and lobular features within a single invasive tumor. Histological grading has proven to be a significant prognostic factor in these patients. Studies have shown that postmenopausal women with mixed ductal/lobular carcinoma have better survival outcomes compared to those with purely invasive lobular carcinoma, indicating a distinct biological behavior [29].

### 4.2. Implications for Practice

This study compared mixed invasive ductal and lobular carcinoma (IDC-L) with invasive lobular carcinomas (ILCs) to assess the overall prognosis, the prognostic role of histologic grade, and the response to systemic therapy. It was found that patients with IDC-L tumors had a better prognosis than those with ILCs, particularly among postmenopausal women, which may impact follow-up strategies. Moreover, although histologic grade failed to stratify the risk of ILCs, it demonstrated significant prognostic power in IDC-L, thus highlighting its clinical utility for guiding treatment decisions in IDC-L. Finally, the disease-free survival advantage of adjuvant aromatase inhibitors over tamoxifen in ILC was consistent in IDC-L.

A comparison of our study cohort’s data with the literature is summarized in Table 5. The age of onset reported in the literature was comparable to our study cohort’s data in invasive lobular carcinoma, papillary carcinoma, and tubular/cribriform carcinoma. In terms of the immunohistochemistry profile, the results were compatible in invasive lobular carcinoma (the majority being ER-positive with only a few being HER2-positive), mucinous carcinoma (the majority being both ER- and PR-positive), and tubular/cribriform carcinoma (the majority being both ER- and PR-positive with only a few being HER2-positive). The result was not comparable in papillary carcinoma, with significantly lower ER- and PR-positive rates in our study cohort when compared with data in the literature. Mucinous carcinoma, papillary carcinoma, and tubular/cribriform carcinoma carried excellent prognosis, while invasive lobular carcinoma showed comparable survival to invasive carcinoma (NOS). All these findings were in agreement with our survival analysis.

### 4.3. Axillary Dissection Rate

Before the introduction of sentinel lymph node biopsy in breast cancer patients by Giuliano et al. in 1994, axillary dissection was regarded as the gold standard for the surgical management of breast cancers [30]. This explains why the overall axillary dissection rate was high in all subgroups, ranging from 56.1% to 92.5%. Over the past decade, advances have been made in the development and standardization of the technique of sentinel lymph node biopsy, with a reduction in axillary dissection rate to 50–80% from 2004 onward in our center.

### 4.4. Limitations of Study

This study evaluated some rarer breast cancer histotypes over a 20-year timeframe. This 20-year span was selected to provide a comprehensive longitudinal assessment of rare breast cancer subtypes, encompassing periods before and after significant advancements in diagnostic imaging, molecular typing, and treatment modalities. This timeframe allows for the evaluation of long-term outcomes and the impact of evolving therapeutic strategies on disease-specific survival. 

It is important to note, however, that this choice also introduces certain limitations. Clinical practices and treatment guidelines have evolved substantially in recent years, and the data may not fully reflect the current state of care. For example, although sentinel lymph node biopsy was introduced back in 1994 [30], the first case of sentinel node biopsy in our unit was performed in 1995 as an experimental procedure; the procedure was subsequently formally launched in 1999. 

Furthermore, changes in screening practices and public health awareness over these two decades might have influenced the stage of the disease at time of diagnosis and subsequent outcomes. In Hong Kong, there was no population-wide breast cancer screening during the 20-year study period, but self-initiated breast cancer screening was found to have increased over the last few decades due to the improved socioeconomic status in the territory. 

This study is a retrospective study, which is intrinsically subjected to weaknesses such as selection bias and misclassification or information bias. However, all the clinical data used were collected from a prospectively maintained database for all the patients under treatment by our unit. Recognizing these factors is essential for interpreting the study’s findings within the appropriate historical and clinical context.

While our conclusions largely resonate with the current understanding of rare tumor histotypes, we acknowledge the constraints imposed by the small sample size inherent in studying less common cancers. This limitation may impede a detailed analysis of tumor characteristics and could potentially influence the robustness of our conclusions. To address this, we suggest that a meta-analysis or systematic review might be necessary for each individual subtype to provide a more comprehensive understanding and strengthen the evidence base. Such collaborative, larger-scale studies could refine and augment our findings, leading to more precise clinical guidelines and improved patient care.

## 5. Conclusions

In summary, the top five rare breast cancers in our database were mucinous carcinoma, invasive lobular carcinoma, papillary carcinoma, mixed invasive ductal and lobular carcinoma, and pure tubular/cribriform carcinoma.

The overall survival did not demonstrate a significant difference in the surgical resection groups, suggesting that the current treatment strategy with complete surgical excision and adjuvant systemic therapy and/or radiotherapy is appropriate. Three of the five most common rare breast cancers, namely mucinous, papillary, and tubular/cribriform carcinomas, showed better disease-specific survival. These three groups were found to have similar characteristics, including an earlier stage of the disease at presentation with less nodal involvement, more hormonal receptor positivity, and less HER2 positivity. Invasive lobular carcinoma and mixed invasive ductal/lobular carcinoma showed comparable disease-specific survival when compared with invasive ductal carcinoma.

This study demonstrated the differences in characteristics and disease-specific survival in different histotypes of breast cancers, which has the potential to influence the management of rare breast cancer subtypes. The distinct clinical presentations and outcomes observed for mucinous, papillary, and tubular/cribriform carcinomas underscore the necessity for histotype-specific treatment strategies. These subtypes demonstrate better disease-specific survival, which could be attributed to earlier stage detection and favorable biological markers, suggesting that current screening and diagnostic protocols might be tailored to enhance early identification and intervention for these rare forms. 

Our analysis also reveals that the unique characteristics of these rare cancers—such as a lower incidence of nodal involvement and higher hormonal receptor positivity—necessitate a more conservative approach toward adjuvant therapy. It is crucial that future clinical guidelines consider these attributes to optimize treatment efficacy. 

Our study highlights the urgent need for further research into targeted therapies for these rare subtypes, as the current understanding of their molecular profiles is limited. Given their distinct molecular biology, research should explore the development and integration of novel therapeutic agents that target specific pathways implicated in these rare cancers. Our study lays the groundwork for future investigations and serves as a call to action for the medical community to refine management approaches, with the ultimate goal of improving patient outcomes for these uncommon yet significant variants of breast cancer.

## Figures and Tables

**Table 1:** Patient cohort.

Total Number of Patients (*n* = 4393)		
Surgical Resection Group (*n* = 4202)		
Histological Type	Frequency	%
Invasive carcinoma (NOS)	3594	85.5
Mucinous	141	3.36
Invasive lobular	139	3.31
Papillary	82	1.95
Mixed invasive (NOS) and lobular	67	1.59
Pure tubular/cribriform	45	1.07
Medullary	39	0.93
Other subtypes	62	1.48
**Palliative Group (*n* = 191)**		
**Histological Type**	**Frequency**	**%**
Invasive carcinoma (NOS)	161	84.3
Invasive lobular	9	4.71
Invasive adenocarcinoma	7	3.66
Mucinous	3	1.57
Papillary	3	1.57
Other subtypes	6	3.14

Note: Percentages are based on the total number of patients in each group. Other subtypes include cases not listed in the main categories.

**Table 4:** Hormonal receptors and HER2 status by histological type.

Variables	Invasive Carcinoma (NOS)	Mucinous	Invasive Lobular	Papillary	Mixed Invasive (NOS) and Lobular	Tubular/Cribriform
Estrogen receptor-positive	50.1%	69.4% * (*p* < 0.0001)	59.1% * (*p* = 0.0373)	52.4%	73.1% * (*p* = 0.0002)	71.2% * (*p* = 0.0049)
Progesterone receptor-positive	40.9%	60.9% * (*p* < 0.0001)	41.8%	50.1%	70.2% * (*p* < 0.0001)	66.8% * (*p* = 0.0005)
HER2-positive	19.7%	5.0% * (*p* < 0.0001)	17.0%	4.9% * (*p* = 0.0008)	3.0% * (*p* = 0.0006)	0%

Notes: * *p* < 0.05, obtained using Pearson’s chi-squared test compared to the invasive carcinoma (NOS) group. Values are expressed as percentages of patients within each histological type.

**Table 5:** Summary of literature review compared to our study cohort’s data.

Variables	Mucinous	Invasive Lobular	Papillary	Tubular/Cribriform
Age				
Cohort data (mean)	54.6 ± 16.3	52.6 ± 11.6	58.2 ± 15.2	54.5 ± 13.3
Literature (mean)	71	63.4 ± 12.7	65.7 ± 13.2	57.0 ± 13.8
Literature (median)	-	-	53	-
ER-Positive				
Cohort data	69.4%	59.1%	52.4%	71.2%
Literature	94%	92.7%	100%	92.9%/100%
PR-Positive				
Cohort data	60.9%	41.8%	50.1%	66.8%
Literature	80%	67.4%	80%	87%/69%
HER2-Positive				
Cohort data	5%	17%	4.9%	0%
Literature	-	7–10.7%	-	2%

Notes: Cohort data represents the study’s findings. The ‘-’ indicates data not available or not applicable in the literature review for the respective category. ER: estrogen receptor; PR: Progesterone receptor; HER2: human epidermal growth factor receptor 2.

## Data Availability

The original contributions presented in the study are included in the article, further inquiries can be directed to the corresponding author.
